# A Treatise on Hysteria

**Published:** 1831

**Authors:** 


Art. VII. *A Treatise on Hysteria.*—By George Tate, M. D., Member of the Royal College of Surgeons in London. Philadelphia: E. L. Carey and A. Hart, 1831. pp. 131.

We know of no disease to which the epithet of "opprobrium medicorum" is more appropriate, than to hysteria. It assumes the mask of so many diseases, and presents itself under such a variety of forms, that while at one time, by its strange fantastical shapes, it exposes the sufferer to the ridicule of her acquaintance, at another, it subjects her to the most excruciating agony that the human frame is ever called upon to bear. Cancer, tetanus, and hydrophobia are so generally acknowledged to be incurable, that our remedies are administered without the hope or expectation of success; and if they are successful in alleviating the gloomy passage to the grave, the friends of the sufferer are abundantly satisfied. In hysteria, on the other hand, we are often mocked with delusive expectations of success, until the spirits of our patients fail under the pressure of disease, and the sickness of hope deferred; and, not unfrequently, is the physician mortified to discover that the constitution of the patient has been rashly sacrificed, while he has been combatting with herculean remedies, some of the symptoms of this ever changing disease. Under these circumstances, every thing which can throw a ray of light upon this mysterious subject, must be acceptable to the profession, and under this impression, we proceed to lay before our readers a brief account of the work before us. We believe, with the author, that this class of diseases is not generally understood, either by young or old practitioners; and are convinced by painful experience that none requires more discrimination and judgment to detect and treat them successfully.

In the 2d chapter, the author treats of the cause of hysteria, and asserts that in all its varieties it has one common origin, which is essential to its appearance; namely, irregular or defective menstruation.

"There is always some deficiency or some depravity of this secretion: it will be be found sometimes altogether suspended; sometimes redundant, or too frequent in its recurrence; sometimes dark and grumous; at others, pale and watery; sometimes it is attended with agonizing pain and sickness. Sometimes, also, Hysteria will take place previously to, and be indicative of, the first appearance of the menses; and sometimes it will occur when there are about to be no more seen. The common conditions, however, under which Hysteria prevails, are catamenial suppression, insufficiency, or depravity." p. 11.

Debility, connected with a deranged state of the stomach, liver, and bowels, certainly predispose to this disease, but the disease, far from being confined to females of a delicate texture, frequently attacks the healthy and robust. On the other hand, if it be true, as the author asserts, that disordered uterine secretion is an invariable forerunner of the disease, the fact goes far towards justifying him in assigning this as the cause of the disease.

Dr. T., looking to the common origin of the various forms of diseases which he describes as hysteria, has avoided all nosological distinctions; only for the advantage of description, marking *hysteria of the first, second, and third degree.*

Chapter III. treats of *Hysteria of the First Degree.* The following description, although deficient in several important, particulars, has the important recommendation of simplicity and conciseness.

"It occurs like other forms of Hysteria, almost invariably between the ages of thirteen and forty-five. It is always attended with some irregularity about the menstrual discharge; and the stomach, liver, and bowels (one or all) are generally out of order. It is characterized by alternate fits of laughing and weeping, starting and screaming, lying still as death, and strugling with gigantic strength. Generally, there is a loud rumbling in the bowels called Clangor Intestinorum, and the Globus Hystericus, as it is called, which is, in fact, mere flatulence, causing a sensation like that of a solid ball rising in the throat and producing a sense of suffocation." p. 16.

The author rejects the copious secretion of pale urine, as being at all a sympton of this disease.

As to the treatment, he represents the antispasmodic treatment as a remnant of an antiquated and false pathology, and speaks of the fetid drugs only as a suitable punishment for those, in whom the disease is the consequence of affectation, or a morbid and vicious sensibility unreasonably indulged. He depends entirely upon brisk cathartics, a mild and nutritious diet, and the use of aloes and iron until the alvine and uterine secretions are properly regulated.

Chapter V., *Symptoms of Hysteria, of the Second Degree.* In this chapter the author gives the essential and diagnostic symptoms of hysteria, by which it may be recognized among the infinite, varied, and contradictory forms which it so capriciously assumes. As this chapter contains the peculiar views of our author, we shall quote from it at considerable length.

"I.—In the first place, menstruation is defective. It always is, and has generally been some months prior to the attack, in some respect, more or less deranged. This derangement is the 'head and front' of the case; the original cause of the disorder; that upon which all the other symptoms are more or less dependent.

"II.—The next circumstance, and the most important of the whole list, whether as regards the discrimination of the disorder, or its treatment, is this:—that, in every case, there is distinct pain upon the application of pressure or of heat, to three or four of the six superior dorsal vertebræ. This is a point upon which I desire to fix the reader's attention; for this spinal affection, whatever its intrinsic quality, is clearly chargeable with most of the curious images, and fantastic forms, that Hysteria is accustomed to put on; and yet, notwithstanding its constant occurrence in these forms of Hysteria, and its frequent existence where there is even a tendency to Hysteric disorder, it is a circumstance that has been overlooked by those who have professed to treat upon the subject, as well as by those who, for the sake of gratifying curiosity, have published detached cases of Hysteria under various other designations.

In other parts of the spine, especially in lumbar vertebræ, pain is frequently complained of foe a long time; but, in the dorsal divisions, no uneasiness is generally felt until pressure is made upon them. Indeed, if the patient be asked if she have pain between the shoulders, she will usually answer, No! and will think it very troublesome to be disturbed for the purpose of exposing the part to examination. Proceeding from the uppermost cervical vertebræ gradually downward, she will smile at the inquiry, "if the pressure of the fingers hurts her?" until you reach the dorsal vertebræ; when her countenance will immediately betray her, and she will shrink from the touch, confessing that the pressure causes a pain which frequently, but not always, goes through either to the chest or to the left side, sometimes to both, and generally oppressing the breath.

I do not mean it to be understood that this pain is always present to the same extent: sometimes the vertebræ are extremely tender, even upon the lightest pressure or the least heat, and at other times they bear a moderate degree of both without much suffering: but the pain is invariably present in greater or less force, bearing some proportion generally to the violence of the Hysterical manifestations; and leaving an aching or soreness in the part for some time after the fingers have been removed.

III.—Another thing to be attended to as a diagnostic symptom, is pain in the left side. This is very peculiar. It is usually situated immediately below the left breast, in a hollow formed between the cartilages of the fifth and sixth, or sixth and seventh ribs; it is generally so circumscribed, that it may be covered by a shilling; and is of the gnawing kind. Occasionally, however, it is most acute, feeling as if a knife were being struck into the spot, and the patient cannot forbear screaming. This pain is complained of for some time before the invasion of the Hysteria. The patient is often observed to incline the upper part of the body to that side, dropping the left shoulder, which relaxes the painful part and affords some relief. The act of raising the left arm above the head, or of bringing the body into a perfectly erect position, is attended with an increase of pain. I apprehend this pain is really seated in the intercostal nerve, although I

have thought it must be situated in the nerves of the heart itself; as it is difficult to account for its perpetual preference for the left side. The right side, certainly, is often not exempt from pain; but in nineteen cases out of twenty, the prominent grievance is in the former; and in the like proportion of instances, I can put a finger on the spot with as much certainty as if it were visibly marked.

I am convinced that many spinal curvatures have arisen solely in consequence of this pain, which is often of very long standing, causing a tendency to lean the body constantly out of the perpendicular line, towards the affected side. In these cases, nothing is more easy than to mistake the effect for the cause. When, therefore, other symptoms supervene, and a medical man is called in, he must not immediately determine the curvature to be the cause of the patient's illness, as it will frequently turn out to be merely a consequence of another disorder. I have seen a young lady confined to her bed for nearly three years by this very mistake; and nothing was gained by it but an increase of weakness.

IV.—Palpitation is another symptom that is almost universally present, to guide the judgment in these disorders; and it is often distressingly violent. A sense of fluttering about the heart is also very common.

In addition to these, there is always pain in the head, in front, or in the occiput, or in both; intolerance of light, which is sometimes so extremely urgent that the patient is easy only in absolute darkness, and generally more or less globus.

Such are the special symptoms which denote Hysteria of the second degree. A suspended, irregular or painful menstruation; pain upon pressure upon some of the dorsal vertebræ; pain underneath the left breast; palpitation or fluttering at the heart; more or less pain in the head; intolerance of light; and more or less globus. These are almost unfailingly combined in every case; and, in the great majority, are accompanied by pain or aching in the arms and legs, and across the loins,—furred tongue—constipated bowels,—and dyspnœa. The pulse is sometimes quick, variable, or intermitting; at other times perfectly natural.

In the treatment of this disease, the author lays down two indications of cure; 1st, The removal of the hysterical evolutions; 2d, The restoration of the uterine and other secretions. Bleeding, he remarks, will rarely do good. It never, in his opinion, relieves the spasms; and, although, in some plethoric individuals, it may do no harm, it frequently produces the most serious injury.

In relation to this remedy, while we admit the necessity of the utmost caution in detecting those symptoms which simulate inflammation in hysterical patients, we cannot but think that the prohibition of blood letting is entirely too exclusive. Hysteria very frequently occurs in very opposite conditions of the system. The cause which, in the opinion of our author, alone produces it, is more frequently brought on by exposure to cold, than by any other morbid agent; and hence the disease frequently partakes of those inflammatory symptoms which so generally accompany the derangements of the system produced by atmospheric vicissitudes. It also frequently occurs in persons of full health and vigor, in whom the sudden suppression of all the excretions must, of necessity, produce such a degree of plethora, as with the nervous excitability which now takes place, will imperiously call for the lancet.

But the author's great remedy is the tartar emetic ointment; in the application of which, he should certainly be allowed to speak for himself.

"Having made a careful examination of the spine, and ascertained the seat of pain, the first thing to be done, is to apply the tartar emetic, either by friction or plaster upon the spot. If the symptoms be urgent,—whether Cataleptic, or choreiform, or tetanic, or hemiphlegiac, or any other,—the application should be carried throughout the whole course of the vertebræ; and this should be done every six or seven hours, until the pustulation is fully developed. The Hysterical symptoms will then begin to yield, and the patient will become calm and sensible: but as the cause of the spinal disorder, viz: the faulty uterine function, is still in operation, it is sometimes necessary to establish the eruption "*iterum iterumque*," in order to secure the patient from a recurrence of the same symptoms. In the meantime, every exertion must be used to improve the condition of the uterine organs.

Upon the discovery of this tenderness between the scapulæ, I have frequently leeched the part, which has always failed to afford any important relief: I have afterwards blistered the spine, without deriving therefrom at best, more than partial benefit; but I cannot too often repeat, that the antimonial eruption exerted always a most powerful influence over the disorder, controlling its various manifestations,—relaxing muscular contractions,—and dispelling the tenderness upon pressure, and the pain on the application of heat." p. 47, 48.

In addition to this, Dr. T. reccommends active purgatives, which seldom fail to bring away dark unhealthy evacuations; and this plan should be pursued at the discretion of the practitioner, until the discharges assume a natural aspect.

The second indication—of restoring a healthy menstruation, must be conducted upon general principles—improving the secretions and correcting the state of the stomach, and other digestive organs. After this, air, exercise, the warm, tepid, or cold bath, with the preparations of iron, constitute the best tonics.

In support of his opinions, Dr. T. details a number of cases drawn from his own practice, and from the Medical Journals; of some of the most interesting of which, we will present an abstract to our readers.

Case 1. A healthy looking country girl came to Dr. Tin, April, 1825, complaining of her eyes, which seemed inflamed, with a great intolerance of light, and a copious flow of tears. Venesection to eight ounces, relieved these symptoms, and rest, low diet, and an active purgative, were prescribed for her. Having been sent for in great haste on the next morning, he found her in great agony, breathing with excessive labor, pressing her hand firmly against her left side, and giving indications of acute pain in that region. The breast sounded well, and the skin was warm, and covered with profuse perspiration.

Upon inquiry, he found that menstruation had been scanty for more than a year, and for fourteen weeks had been entirely suppressed, and that she had suffered from palpitation and pain in the side. Pressure upon the four uppermost dorsal vertebræ produced pain, which was referred to the left side, and scrobiculus cordis. These symptoms led the author at once to the opinion that the disease was hysterical. At the instigation of her friends she was again bled, without relief; but Dr. T's principal reliance was upon the tartar emetic ointment, and upon active purgatives. These means were assiduously employed. On the first day, many offensive evacuations were procured, but without relief. On the third day, the eruption began to appear, the symptoms were very much mitigated, and on the next day, were entirely removed.

From this time she gradually recovered her strength, but the catamenia did not return, and in six weeks she had a new attack. This attack also yielded to the antimonial plaster, although a blister had been ineffectually applied to the whole course of the spine. A third application of the ointment was necessary to remove the tenderness completely; and having since menstruated, she has enjoyed uninterrupted health.

Case 2. Miss W., aged 15, after several days of slight indisposition, awoke in the morning, after a tranquil night, with a train of symptoms resembling tetanus. She was resting on the head and heels, the body being bent into the form of an arch, the arms and legs being flexed and rotated inwards, and the fingers and toes being violently closed. She was perfectly sensible, and complained of great heat and pain in the head and intolerance of light, and squinted very much. Pulse 110, skin hot and covered with moisture. Dr. T. examined the spine, and the instant pressure was made on the dorsal vertebræ, the patient complained of pain. By the free action of purgatives, the head and the respiration were somewhat relieved, but the spasm continued unabated, until the eruption was produced by the antimonial ointment, and in twenty-four hours after this, all the symptoms had vanished. Once or twice the tetanic symptoms returned, but they were promptly relieved by a second application of the ointment. In five weeks from the attack, she menstruated, and has since remained well.

Case 3. In January, 1825, Dr. T. saw, for the first time, Miss L., aged 19 years, who had been in delicate health for nearly four years. For several days she had had an acute pain in the side, increased by inspiration, but relieved by pressure, and so severe as to require her friends "to hold her sides for hours together." A short time before Dr. T. saw her, she fell back in her chair, apparently lifeless. Respiration would, for a time, be, apparently, perfectly suspended, then a deep inspiration followed by rapid breathing, would, be followed by a death-like stillness. All this time her pulse remained regular, except occasional violent palpitations. When she recovered for a short time, she conversed rationally, but complained very much of her head, side, and chest, and of great sensibility to the light.

Dr. T. having convinced himself that there was no disease of the heart, examined the spine, and found that pressure on the dorsal vertebræ produced pain, which, passing to the side and the pit of the stomach, increased the difficulty of breathing.

The catamenia which had appeared a few days before, was unusually scanty and dingy. The antimonial plaster was applied to the back, and when the eruption came out, the symptoms very soon subsided. Once or twice there was a slight relapse, but the symptoms were soon dissipated, and she has since enjoyed tolerable health.

Case 4. E. M. aged about 20, of a delicate constitution and feeble health, had suppression of the menses for several months. When Dr. T. saw her, she was in a state of insensibility, and affected with the most violent convulsions; pulse active and bounding. Blood was drawn without relief. Active purgation relieved the convulsions, but the insensibility continued unabated.

The tartar emetic ointment was applied, and when sensibility was in some degree restored, the spine manifested an unusual degree of sensibility, extending to the left side, and producing an excessive degree of tenderness under the ribs on the right side. These symptoms were all relieved by the tartar emetic ointment; the menses were brought back by the usual combination of aloes and iron, and the patient has since enjoyed better health than at any former period of her life.

Case 6 was accompanied with violent convulsions, of a choreal character, lasting for hours, with complete insensibility. In the intervals, she complained of the globus, intolerance of light and noise, palpitation, pain in the left breast, in the head, and down the arms. The menstrual discharge had been for several months of a dark and depraved character, and attended with severe pain in the loins. Bleeding, leeching, blistering, active aperients, with ammonia, valerian, and other antispasmodics, had been tried without any other effect than to increase her distress. The antimonial ointment was applied—in three days the eruption came out, and the unpleasant symptoms vanished.

The author, in Chapter VII., gives a transcript from the Med. Chirurg. Review, of nine cases of anomalous disease, treated by different practitioners under different names, or under no name at all, which he supposes to have been cases of hysteria. It would be no slight degree of labor, to recapitulate the various symptoms which are here recorded; it is sufficient to say that the catalogue would include almost every symptom of every inflammatory and every nervous disease. The catalogue of remedies is less formidable only than the symptoms.—The lancet, leeches, cups, purgatives, blisters, setons, issues, acupunctuation, tonics, alteratives, and the whole class of emmenagogues, every thing, in short, but the antimonial ointment, to the spine. These cases had the common symptom of suppressed or imperfect menstruation, and if any thing were wanting to convince us of the justness of the author's views in referring them to this common cause, we should have it in the tenacity of life which these patients display, and the manner in which they bear up under the violence of the disease and the severity of the treatment.

One of these cases which terminated fatally, we shall present to our readers, since hysteria so seldom proves directly fatal, that the *post mortem* examination is both interesting and important.

"The following is from No. XIII, New Series, Page 146. It is headed " *Chorea fatal.*"

"E Smith, aged 17, was admitted into the Middlesex Hospital, under the care of Dr. Hawkins, on the 5th of September, 1826. She had just recovered from a severe attack of rheumatism in her knees and shoulders, which lasted seven weeks. A fortnight before her reception, she was seized with involuntary convulsive movements in the legs, arms, and neck. These had continued ever since with great violence. *The catamenia had been suppressed four months.* She had head-ach, thirst, pain in the back, pulse 96, tongue loaded, bowels constipated. She was actively purged without success. The calomel, senna, and turpentine, always dislodged dark and copious motions; but produced no alleviation of the convulsive spasms, which were like those of hydrophobia. She could not hold her head quiet for an instant, and the grinding of her teeth was so violent as to force one of them from its socket. The convulsions were uninterrupted, except by short intervals of broken sleep. Her intellects were unimpaired. She was put into a warm bath, which aggravated her convulsions, produced great irritation, and inflammatory symptoms,

Sixteen ounces of blood were drawn; it was *inflamed.* She was bled again the next day,—still very little alleviation of the spasms. Musk was then tried without effect. Camphor and opium procured her some sleep: after taking them the second time, she slept soundly; awoke, and soon afterwards expired on the 13th of September: being eight days after her admittance.

"*Dissection.*—No morbid appearance could be found in the brain. There were tubercles in the lungs, and earthy concretions in various parts. Adhesions between the liver and adjacent parts, intestines healthy in appearance; omentum and mesentery studded with numerous cysts: some containing a black semifluid matter, others calcareous depositions. Several large concretions in the pancreas. *The uterus was rather large and vascular, and the lining membrane of its body and fundus highly injected. The Fallopian tubes and ovaries contained a good deal of the black matter above-mentioned.*"

In all these cases, attended by eminent physicians, it is remarkable, that although suppressed menstruation preceded the other symptoms, it never occurred to their minds as the cause of the disease; and it is perhaps not less surprising, since irritation of the spinal marrow has of late attracted much attention, as the cause of many anomalous forms of disease, that the spine should never have been subjected to examination, the more especially, as many of the symptoms would naturally be referred to irritation of the brain, or spinal marrow.

Chapter VIII. treats of what the author calls *hysteria of the third degree.* This distinction, however, must be understood to relate only to a more insiduous and protracted, consequently a more obstinate and hopeless form of *the same disease.* The same condition of the uterine functions precedes the cases of this kind, and the same spinal irritation attends them, but they generally come on gradually, without the eruption of the more violent symptoms of hysteria, so that the true origin of the disease is not suspected, until the patient sinks into a state of deplorable debility, or by the sudden accession of more violent symptoms, the attention of the practitioner is directed to a more correct pathology. From these cases, the usual marks of hysteria are seldom absent, and they may, therefore, generally, be recognized, if the attention of the physician be not too exclusively directed to symptoms which seem to occupy a more prominent station in the train of diseased action. Like other forms of hysteria, too, they are seldom dangerous, unless maltreated; but they render the patient as miserable as the absence of enjoyment and the entire destitution of hope can make her.

The following is the author's account of the disease in its most protracted and inveterate form—a general description which he afterwards fills up by a report of cases.

"As before stated, in the most tedious form of Hysteria, menstruation is always more or less faulty at the onset, and as the case advances, this becomes suppressed altogether, or is performed very sparingly, perhaps only once in many months, and then with great pain. Where this function is quite suspended, there is, generally, neither any periodical pain nor any sensation, to show that nature had not forgotten this customary duty. Shortly afterwards, the patient becomes weak and desponding, loses her appetite, and the bloom from her cheeks. She has still nothing particular to complain of, and, generally, keeps up her flesh, although it has every appearance of relaxation. If a medical man sees her now, he will find her with a moist and tremulous tongue; being foul at the root, and having the papillæ, at that part, larger than natural and like little tubercles; with a tainted breath; depraved taste; little or no appetite; with a weak languid pulse; with a sickly yellowish complexion; black or clay-colered alvine secretions, and the urine highly colored and scanty. In a little time, she will have pain under the left breast; which is increased by deep inspiration, and by reclining upon that side,—sometimes pain also in the right side, palpitations, flutterings, sinkings, and together with these, there will be pain upon pressure in one or more parts of the spine; first of all in three or four of the dorsal vertebræ; generally, also, in the lumbar, and if the case be very lasting, it sometimes extends up to the very summit of the cervical portion. In such cases, the headachs are intolerable; being, in some instances constant, in others interrupted, but always violent. The pain is often continued down the arms and into the legs; the extremities are generally clammy and cold. In the midst of all this, the patient is not much reduced in flesh, and for a considerable time, it is not sensibly diminished. As the disease advances, a number of anomalous pains of a neuralgic character, become associated with the other symptoms. Thus, if pressure be made upon the supra or infra-orbital nerves, upon the inferior maxillary, &c., as they issue from their foramina, considerable pain is produced, but I never found these spots complained of, in the absence of such pressure. It is not, however, the facial nerves that are alone implicated, for almost every nerve in the body becomes at the same time, endued with a similar increase of sensibility. This sort of neuralgic affection is seldom observed untill the case is far advanced, and has become equally inveterate and puzzling." p. 90—92.

For the remainder of the cases detailed by the author, we must refer the reader to the work itself. From one only, remarkable for its long continuance, extensive complications, and terrible mismanagement, we shall make a few extracts. This patient came under Dr. T's care, in March, 1825, for an illness of ten years' standing. Twelve different practitioners had attended her with an equal want of success. Her illness commenced shortly after she had menstruated the first and only time.

When I first saw her, she had just returned from a watering place, was so weak that it was with difficulty she could walk across the room, and was obliged to be carried to and from her apartment. Her complexion was sallow, her lips bloodless, pulse small and quick, tongue loaded with a thick, yellowish-brown, moist secretion,—bowels torpid, —dejections of various hues, but always unhealthy—the water uniformly clear and straw-colored. There was a fixed and lancinating pain in a hollow, between the cartilages of the fifth and sixth ribs of the left side; pain under the margin of the ribs of the right side; considerable difficulty of breathing, and frequent violent palpitations. The head-aches were almost incessant, and often nearly distracting by their violence. There was pain upon pressure throughout the cervical and dorsal vertebræ; and pressure between the shoulders, aggravated the dyspnœa. She was sometimes seized with an uncontrollable vomiting, which lasted seven or eight days together; at which times, not a spoonful of cold water would remain upon her stomach; these attacks were ultimately tranquillized by opiate suppositaries, leaving her strength completely prostrate. She scarcely ever closed her eyes to sleep, although her sufferings were so great, that she was lying in a recumbent posture, at times for days and nights together, with her eyes shut in silent agony. She appeared literally not to eat any thing. *She had not menstruated since the beginning of her illness, when she was near sixteen years of age.*

Dr. T. was informed, that about four years before this, a physician had examined the condition of the spine, but paid very little attention to the tenderness in that region, referring it to the position in which she lay. Dr. T. after trying ineffectually to remove the pain in the side, referred the whole disease to hysteria, and applied the antimonial plaster, which produced so much distress, and did so little good, that she obstinately refused to have it renewed. A mild general treatment was then adopted, consisting of aperients and carbonate of iron. A small issue was also established over the seat of the pain, but without any good effect. Gradually, her condition was very much improved, and the catamenia returned once, sparingly, but never again.

"In this state, she went to the neighborhood of Bath; and, under the direction of an eminent surgeon, was bled once a fortnight. When it was first proposed, she wrote to consult me upon the subject; and I strongly advised her on no account to submit to so life destroying a treatment. However, the surgeon told her, that he had once effected a cure, by this practice, *in a similar case;* and it accordingly was put in force. For twenty-four successive fortnights, this was continued, at the end of that time, I met the young lady in Cheltenham by appointment. She had not been bled for many weeks, as, upon the last occasion that this operation was performed, a long and deep syncope ensued, from which she was with difficulty recovered. She had now an irregular pulse —violent palpitations—œdematous legs, even to the knees—cold extremities—shortness of breath—and a countenance indicative of exhaustion and distress. the left breast was very much wasted, as were also some of the muscles on the side of the chest: producing a degree of deformity that was evident through her clothes. I earnestly entreated her to subject herself to no more such ruinous experiments; but to take wholsome food—to take as much exercise as her strength would bear, short of fatigue; to take no medicine but a tonic-aperient pill; and to use the shower bath twice, and the warm hipbath three times a week. She then proceeded to Leamington, where she has followed these directions. Her health improves, but the wasting, and numbness, of the left breast and side, are making gradual progress. There appears little hope of her complete recovery, although she has already endured little short of a quarter of an ordinary life of diversified suffering." pp. 98, 99.

Comment upon this case is unnecessary. That the physicians entirely mistook its nature, and that the treatment originally employed was every thing but judicious, all will admit. The remaining cases present the same mortifying account of mistaken views and pernicious practice. The author has an additional chapter upon various other forms of disease, under which hysteria masks itself—abdominal enlargement sometimes mistaken for pregnancy, dropsy, pains resembling rheumatism, or the hip-joint disease, &c. &c. These cases may generally be distinguished by their bearing the general physiognomy of hysteria, by the slight inroads made upon the constitution compared with their violence, and by the obstinacy with which they resist every mode of treatment that is not founded upon their hysterical origin.

Upon taking leave of the work, we cannot avoid congratulating the profession upon the prospect of having a new ray of light thrown upon this obscure, intricate, and troublesome disease. Whether tenderness of the spine and mismenstruation are invariably present, we are not prepared to say. Respecting the latter symptom, too many physicians are in the habit of making their investigations in too careless a manner. We have met with a few cases of hysteria in persons laboring under prolapsus uteri, who were either nursing, or who menstruated regularly; and in addition to this, persons whose testimony is entitled to the utmost confidence, have assured us that the position advanced by the author is utterly untenable. We feel inclined to believe, therefore, that hysteria, or many of its symptoms, may be produced by other forms of uterine disease, than the one alluded to by the author, but that it is always connected with an unhealthy condition of the uterus, we have no doubt.

With regard to pain in the spinal column, we believe, from cases that have lately fallen under our own observation, and from many others in which investigation has been made at our request, that this symptom is very generally, if not universally present, though perhaps not uniformly in the dorsal vertebræ.

